# Powder to become crystal clear

**DOI:** 10.1107/S2052252515004017

**Published:** 2015-04-30

**Authors:** Quan Hao

**Affiliations:** a Physiology, L04-48 Laboratory Block, University of Hong Kong, 21 Sassoon Road, Hong Kong

**Keywords:** serial crystallography, polycrystalline samples, multiphase

## Abstract

A simulation by Zhang *et al.* [**IUCrJ** (2015), **2**, 322–326] has demonstrated that utilizing serial crystallography may turn one-dimensional powder diffraction from multiphase polycrystalline samples into three-dimensional single-crystal diffraction patterns.

The development of X-ray free electron lasers (XFELs) has opened up new opportunities for experiments that seem impossible now to become a reality in the near future. One of the new capabilities of XFELs is to collect single-crystal diffraction data from randomly oriented sub-micron-sized crystals using serial femtosecond crystallography (SFX) (Chapman *et al.*, 2011 ▶).

Many important materials, such as zeolites, are polycrystalline (powders) and cannot be grown as single crystals. Furthermore, different types of samples (multiphase) may be mixed during production of a material; for example, zeolite NU-87 may occur as an impurity in zeolite TNU-9 (Hong *et al.*, 2007 ▶). X-ray diffraction from such samples will usually result in a one-dimensional powder pattern (Fig. 1 ▶, left). Because of the relatively large molecular size (76 non-hydrogen atoms in the case of TNU-9), the powder diffraction pattern from a zeolite can be difficult to interpret (Gramm *et al.*, 2006 ▶). The powder diffraction pattern from a mixture of TNU-9 and NU-87 would be impossible to process.

Powder samples are essentially a mixture of sub-micron-sized (typically 100 nm) single crystals. The latest sample handling techniques, such as liquid jet injectors, can deliver the crystals to the beam one at a time and the extremely intense XFEL beam can capture a diffraction image of each crystal in a sub-nanosecond time scale (Spence *et al.*, 2012 ▶). In this issue, Zhang *et al.* (2015 ▶) have proposed the use of serial crystallography to turn powder diffraction into single-crystal diffraction (Fig. 1 ▶). A test has been performed using simulated diffraction patterns. The test sample is a mixture of zeolites TNU-9 and NU-87 with crystal grain sizes as small as 100 nm. X-ray diffraction snapshots by SFX were simulated and processed using the program suite *CrystFEL* (White *et al.*, 2012 ▶). Identification according to the primitive unit-cell volume determined from individual snapshots was able to separate the whole set of snapshots into two subsets, which matched the two zeolites in the sample. Monte Carlo integration in *CrystFEL* was then applied to them separately. Two sets of three-dimensional single-crystal diffraction intensities could then be derived. The crystal structures of the two zeolites were solved using the direct methods program *SHELXD* (Sheldrick, 2008 ▶) with default parameters.

Turning one-dimensional diffraction from polycrystalline (powder) samples, particularly from multiphase samples, into three-dimensional single-crystal diffraction patterns has long been regarded as a difficult, if not impossible, task. Zhang *et al.*’s proof-of-principle study has demonstrated that with the latest XFEL and sample delivery technology, single-crystal diffraction patterns can be collected from multiphase polycrystalline samples, processed, and then the molecular structures can be solved *ab initio*. This technique promises to open up new avenues for the study of many important polycrystalline materials that cannot be analysed by conventional X-ray powder diffraction methods.

## Figures and Tables

**Figure 1:**
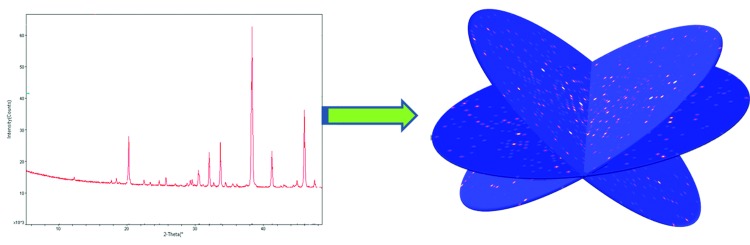
A one-dimensional powder diffraction pattern seen using conventional methods (left) may potentially be analysed as three-dimensional single-crystal patterns using serial crystallography (right).
